# Status and Best Management Practices of Potato Early Dying Disease in New Brunswick, Canada

**DOI:** 10.3390/biology14050514

**Published:** 2025-05-07

**Authors:** Khalil I. Al-Mughrabi, Rene Poirier, Salah Eddin Khabbaz

**Affiliations:** Potato Development Centre, Department of Agriculture, Aquaculture and Fisheries, 39 Barker Lane, Wicklow, NB E7L 3S4, Canadasalah_edk@yahoo.co.uk (S.E.K.)

**Keywords:** potato early dying disease (PED), *Verticillium dahliae*, soil-borne pathogens, root-lesion nematode, fumigation, management

## Abstract

Potato early dying (PED) disease complex is often called the *Verticillium* wilt of potato and is considered one of the most economically devastating diseases of potato worldwide. The severity of the disease greatly increases with the association of the soil-borne pathogens *Verticillium dahliae* and *V. albo-atrum* and the root lesion nematode (*Pratylenchus* sp.). Recently, an increase in wilt disease symptoms and a sharp decline in marketable tuber yield were observed in New Brunswick (NB), Canada. A survey of 71 fields, along with eight fumigated and eight non-fumigated fields, was carried out to determine and quantify nematodes and *Verticillium* in the soil. Techniques used included plate counts for *Verticillium* (CFU/g soil), real-time qPCR (RT-qPCR) for *V. dahliae* (cell/g soil), and nematode identification and counts (# of nematodes/kg of soil). The survey results of the 71 fields revealed that 55 fields had *Verticillium* sp. ranging from 2 to 66 CFU/g of soil by the plate method, and 68 fields had high *V. dahliae* ranging from 261 to 27,471 cell/g of soil by RT-qPCR method. All fields had high numbers of root lesion nematodes ranging from 560 to 14,240 nematodes/kg of soil. There was an uneven distribution of PED incidence in potato fields at various locations of NB. Fumigation with Chloropicrin significantly reduced the numbers of root lesion nematodes by 34.1–99.0%, *Verticillium* sp. CFU/g of soil by 50–100%, and *V. dahliae* cell/g soil by 38–91% in the eight fumigated fields. The management of the PED complex with various disease management products under field conditions was also studied in a field plot trial setup. The nematicide Velum applied in-furrow at the recommended label rate decreased the numbers of root lesion nematodes by up to 66% compared to other products. The combination of both Velum + Aprovia and the application of ammonium-lignosulfonate significantly reduced *V. dahliae* by 190.55% and 274.24%, respectively, compared to other products. The fungicide Aprovia applied in-furrow at the recommended rate for the management of *Verticillium* wilt significantly reduced *Verticillium* sp. CFU/g of soil in treated soil by 73.3% compared to Velum, Mustgrow, Senator PSPT, Vapam, ammonium-lignosulfonate, Nimitz, and the untreated control. Disease management products increased potato marketable yield by 27.38–97.74%. The results of this study suggest that the root lesion nematode and *V. dahliae* have a ubiquitous distribution in the fields cultivated with potatoes in NB. The co-infection of potato by both *V. dahliae* and the root lesion nematode can greatly increase the severity of PED. Fumigation with Chloropicrin significantly reduced the levels of root lesion nematodes and *Verticillium* in all fumigated fields. Management practices of PED using the fungicide Aprovia, the nematicide Velum, and a combination of both Velum + Aprovia had the greatest effect in reducing the population density of the root lesion nematode and *Verticillium dahliae* in soils of commercial potato fields in New Brunswick.

## Simple Summary

Yield reduction due to potato early dying (PED) in severely affected fields can exceed 50%. The aim of this research was to determine the incidence and abundance of *Verticillium dahliae*, *V*. *albo-atrum*, and nematode propagules in soil samples collected from the major potato cultivation areas in New Brunswick, Canada; identify and quantify the density of *Verticillium* microsclerotia in the soil using both soil plating and molecular methods to better understand the specific pathogens present and to assess the efficacy of various management measures on PED; assess the effectiveness of fungicides and nematicides and soil amendments in managing PED; and perform a field-scale evaluation of the effectiveness of fumigation to manage PED. This research has highlighted that the populations of root lesion nematodes, which were present in every field tested, were at much higher populations than previously thought, and that 77% of these fields also had *V. dahliae* present, providing a significantly challenging management issue. Fumigation with Chloropicrin was effective in reducing both nematode and *V. dahliae* populations. Velum reduced both *V. dahliae* and nematodes, with Aprovio working well to reduce *V. dahliae*. The mustard seed meal and bio-stimulant/fertilizer also provided a reasonable decrease in *V. dahliae* soil propagules.

## 1. Introduction

Potato early dying (PED) is also known as early die, early maturity wilt, and *Verticillium* wilt [1]. *Verticillium* wilt is most important in temperate regions, occurs less frequently in the subtropics, and is rare in tropical regions of the world [2,3,4,5]. This disease is endemic in many fields with a long history of potato (*Solanum tuberosum* L.) production. PED results in premature vine senescence and can reduce potato tuber yield by 30% or higher. In North America, yield reduction in severely affected fields can exceed 50% [1,6,7]. The disease is caused by either *Verticillium dahliae* Kleb. or *V. albo-atrum* Reinke and Berthold, which are pathogenic to potatoes and have been reported to occur in Canada [8,9,10,11]. Both species invade xylem elements, disrupt water transport in plants, and cause vascular wilt in a variety of hosts. *Verticillium* species are soil-borne fungi that can survive in the soil as resting structures, agglomerates of thick-walled clumps of melanized cells called microsclerotia (MS), without a host for at least 14 years [4,12,13]. MS density is one of the factors affecting disease incidence and severity [13,14,15,16]. Synergistic interactions of the main causal agent of PED (*V. dahliae*) and/or other pathogenic fungi (*V. albo-atrum*, *Colletotrichum coccodes* (Wallr.) Hughes) and the root-lesion nematode (*Pratylenchus penetrans* (Cobb) Chitwood and Oteifa) have been shown to severely increase PED incidence and to reduce yield [17,18,19]. The PED disease is characterized by a general decline of plants 4 to 6 weeks earlier than normal maturity. The disease first appears as interveinal chlorosis on the leaves of individual or groups of plants scattered among healthy-appearing plants. Wilting and necrosis then follow and progress acropetally from the stem base. The symptoms observed are an uneven chlorosis of the lower leaves, flagging, unilateral leaf wilting, or death. Stems eventually become necrotic and senesce prematurely and may remain upright. A tan vascular discoloration may occur near the stem base [20,21,22,23]. Vascular browning may develop in the tubers of some cultivars [24,25]. Plants across an entire field could die over a several-week period.

Management of the *Verticillium* wilt pathogens is very difficult due to its wide host range and the production of MS that can persist in the soil for several years [7,26,27]. Several studies have shown promising results of the effect of organic soil amendments, including animal and green manures, disease-suppressive crop rotations, bio-fumigation with mustards, and chemical fumigation with metam sodium and Chloropicrin in managing *Verticillium* wilt [28,29,30,31,32,33,34,35]. While effective, chemical fumigation represents a significant cost for growers and may also adversely affect soil health; it is also a practice that is scrutinized by the public and consumers. Fumigation represents a practice increasingly considered to be non-sustainable, although it significantly increases tuber yield and gives the economic control or suppression of *Verticillium* severity. Consequently, it is necessary to consider alternatives to chemical fumigation for the management of PED [7,32,36,37].

New disease management products have become available. The fungicide Aprovia is registered in Canada for the management of soil-borne diseases of potatoes, including *Verticillium* wilt. The nematicide Velum Prime is registered for the management of nematodes, including the root lesion nematode. Limited assessment of these products has been performed in Atlantic Canada (AC). A promising option for the management of PED, especially where *P. penetrans* is present, including AC, is bio-fumigation using cruciferous crop residues such as high glucosinolate mustard varieties, marigolds, and pearl millet, all of which have shown good potential to reduce nematodes [33,34,35,38]. Practices to manage PED may have a strong influence on soil health. In addition, the severity of PED symptoms may be reduced in healthy soils despite elevated pathogen populations. For example, in Manitoba, soils with high organic matter content may not show PED symptoms and yield reduction despite the very high levels of *V. dahliae* in the soil (>200 CFU/g). There is a poor understanding of how management practices of PED influence soil biological communities and indices of soil health. Particularly, chemical fumigation is known to reduce beneficial microbial communities and aggravate diseases in subsequent potato crops. In our most severely affected PED soils, a combination of chemical fumigation to reduce *Verticillium* and nematode pressure followed by soil health building (e.g., compost, green manures) is a promising and practical option to increase yield and avoid dependency on fumigation. The aim of this study was to (1) determine the incidence and abundance of *V. dahliae*, *V. albo-atrum*, and nematode propagules in soil samples collected from the major potato cultivation areas in NB; (2) identify and quantify the density of *Verticillium* MS in the soil using plating and molecular methods (PCR and RT-qPCR) that can assist in better understanding the specific pathogens and assessing the effect of various management measures on PED; (3) assess the effectiveness of registered fungicides and nematicides and soil amendments in managing PED in commercial potato fields in NB; and (4) perform a field-scale evaluation of the effectiveness of fumigation to manage PED in commercial potato fields in NB.

## 2. Materials and Methods

### 2.1. Soil Survey for Verticillium Species and Nematodes in NB Potato Fields

**Figure 1:**
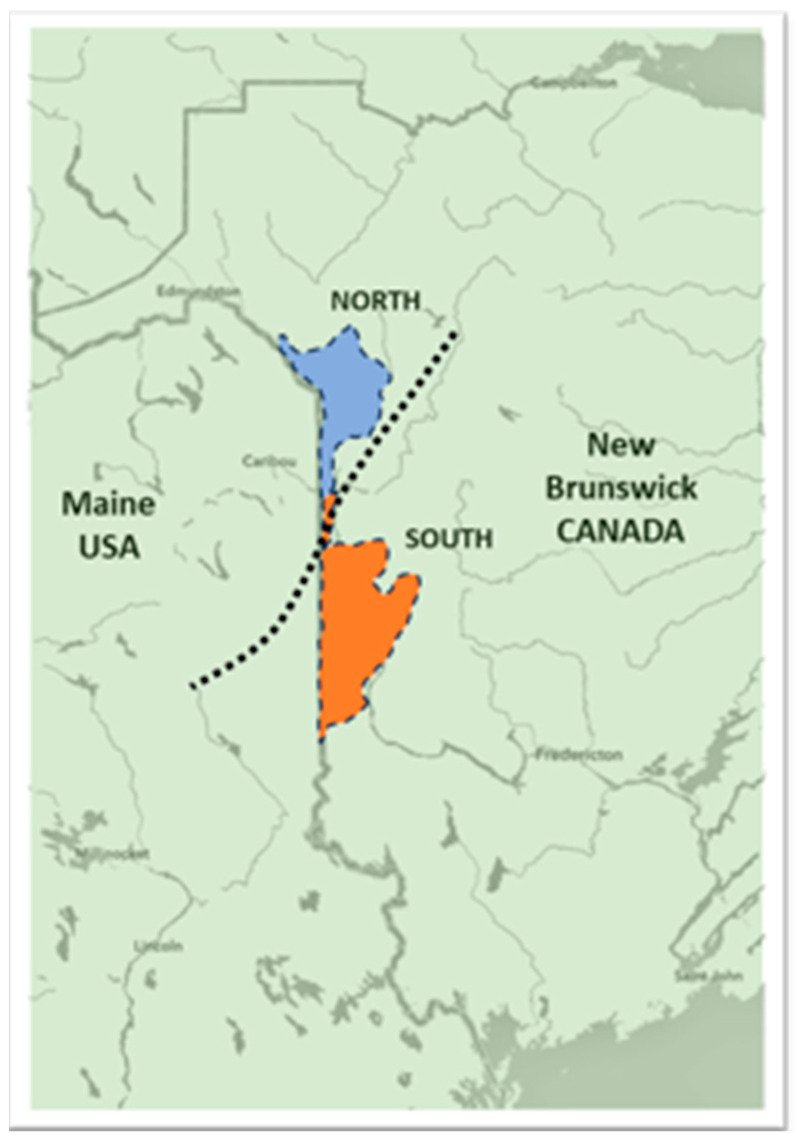
Location of 71 fields surveyed in the potato belt area of New Brunswick, Canada. Northern and southern zones of potato growing region are divided by natural barrier highlands around Perth-Andover, Tobique River (dotted line).

A field survey was conducted to investigate the nature and severity of PED in commercial potato fields in NB. Seventy-one (71) fields were soil-sampled in the fall of 2017 after the potato crop was harvested. Fields were located in the northern and southern parts of the potato belt (Figure 1), representing a range of soils and cropping systems. Soil samples were used to identify, determine, and quantify *V. dahliae* and *V. albo-atrum* species. This assessment was performed at the Potato Development Centre in Wicklow, NB, using plate count technique (CFU/g soil) and at the Agricultural Certification Services Inc. in Fredericton, NB, using RT-qPCR (cell/g soil). Pathogenic nematode identification to genus level and counts (# of nematodes/kg of wet soil) were conducted at the Agriculture and Food Laboratory of the University of Guelph in Guelph, Ontario.

Soil samples were collected from 0 to 30 cm depth in each field using a soil core probe (2.5 cm diameter) (Jeanoko, Shenzhen, China). The top 2–5 cm of soil was discarded, and the rest was placed in a clean plastic pail and thoroughly mixed. Multiple subsamples were collected following a zigzag pattern at each site to ensure an accurate representation of *Verticillium* spp. and nematode distribution. The number of subsamples collected was dependent on field size (1 sample per 3 m). Approximately 2 kg of soil was obtained from each field, divided into two labeled and sealed soil sampling bags, and maintained at 4 °C until sample analyses were conducted. Half of each soil sample was stored in a refrigerator (5–6 °C) until shipping to the Agriculture and Food Laboratory at the University of Guelph for nematode identification and counts. These samples, in accordance with the University of Guelph soil sampling protocol, were not dried or sifted prior to shipping. The other half of each sample was allowed to air dry for 1–2 weeks at room temperature until the soil was dry enough to be sifted through a sieve (250 µm) (Uline, Toronto, ON, Canada). Sifted soil (10 g) from each sample was transferred into a labeled 20 mL plastic vial and shipped to Agricultural Certification Services Inc. for molecular analyses. The rest of each soil sample was analyzed at the Wicklow potato pathology laboratory for *Verticillium* count. The same soil sample protocol was also used for analyzing the soil samples collected before planting and after harvest (spring and fall, 2017) to evaluate various disease management products against nematodes and *Verticillium* [2,39].

### 2.2. Nematode Extraction and Quantification

Nematodes were extracted from 71 composite oil samples representing 71 fields in NB using the Baermann pan method. Specifically, mixed soil (50 g) was placed onto facial tissue (preferably 1-ply) and situated on a mesh layer within a pan. Distilled water was added until a thin layer of water formed at the bottom of the pan. The pan was then placed inside a plastic bag and left undisturbed at room temperature for one week to allow the nematodes to migrate through the tissue. Following the one-week period, water collected in the pan was poured into test tubes where nematodes were allowed to settle for a minimum of three hours. After nematodes had settled at the bottom of the test tube, overlying water was removed, and the nematode-containing water was poured into a Plexiglass counting dish (University of Guelph, Guelph, ON, Canada). Using a dissecting microscope (Carl Zeiss AG, Baden-Württemberg, Germany), nematodes were then counted and identified [40].

### 2.3. Plate Counts

All soil samples were processed within 1–2 weeks of receipt. A thin layer of soil was spread onto a piece of paper, and any lumps were broken up. Prior to plating onto Sorensen’s NP-10 Media (Millipore Sigma Canada Ltd., Oakville, ON, Canada), the soil was air dried at room temperature (25 °C) for 2–4 weeks (depending on soil moisture content) to kill conidia and mycelial fragments of *V. dahlia* [41]. The soil was gently passed through a #10 mesh sieve (2 mm) (Uline, Toronto, ON, Canada) to remove any stones or plant debris. A 10 g of thoroughly mixed soil sample was stored in a small zip-closure bag at 4 °C to use in soil plating as described below.

#### 2.3.1. Suspension Solution

A suspension solution was prepared a minimum of one day prior to plating the soil samples. This solution helps in suspending the soil particles in the flask. One flask was used per soil sample. A 0.1 g of Bacto agar (Millipore Sigma Canada Ltd., Oakville, ON, Canada) was added to 100 mL of deionized water in a 250 mL Erlenmeyer flask and then shaken gently. The top of the flask was covered with aluminum foil, and the flask was then autoclaved for 15 min at 121 °C. All flasks were shaken gently after they were autoclaved to avoid agar solidification. All flasks were allowed to cool overnight or longer and then stored at room temperature [41].

#### 2.3.2. Sorenson’s NP-10 Semi-Selective Medium

To favor the growth of *V. dahliae*, a semi-selective carbon source, such as pectate (polygalacturonic acid) that can only be metabolized by a few other fungi, was added to the agar medium. In addition, Tergitol NP-10 (NPX) (Millipore Sigma Canada Ltd., Oakville, ON, Canada) was used to restrict fungal colony size. Antibiotics were used to prevent bacterial growth [42,43].

#### 2.3.3. Plating Soil

A 10 g soil sample was added to a flask containing 250 mL of water agar and was then agitated on a platform shaker at 50–60 rpm for 2 min. A 1 mL aliquot from the middle of the suspension was taken using a sterilized pipette tip after cutting 3–5 mm to widen the orifice to allow larger soil particles to be delivered onto the surface of a Petri dish containing Sorenson’s NP-10 media. Ten plates per soil sample were used. Under a sterile laminar hood, a 1 mL aliquot was spread as evenly as possible using a sterilized plastic spreader. Plates were covered half to three-quarters with their lids until the water evaporated and the surface of the plate was dry. Ten plates for each soil sample were wrapped together in plastic wrap and then incubated upside down in the dark (to avoid chlortetracycline becoming fungistatic) at room temperature for 3 weeks [41].

#### 2.3.4. Colony Identification and Count

After 3 weeks of incubation, each plate was run under a very gentle stream of cold water. A gloved finger was used to gently rub the surface of the plate to eliminate and remove any surface mycelia and soil particles. The excess water was then drained. The entire surface of each plate was scanned under a dissecting microscope, and the developed colonies of *Verticillium* containing newly formed MS were counted. Colonies generally appear as black lines of MS radiating from a central point below the surface of the agar. Absence of yellow pigment or short conidia and the presence of microsclerotia are the morphological characters of *V. dahliae* as described by Inderbitzin et al. [4]. The inoculum level was expressed as the number of colony-forming units (CFUs) per gram (weight or volume) of soil, which is equal to the sum of colonies (MS) on the ten plates.

### 2.4. Pathogen and Nematode Identification

Soil samples were used to identify *Verticillium* species present in soil. Identification of *Verticillium* spp. was performed at the Potato Pathology Laboratory in Wicklow, NB, using traditional methods and at the Agricultural Certification Services Inc. in Fredericton, NB, using conventional PCR and sequencing techniques.

Pure cultures of *V. dahliae* and *V. albo-atrum* were grown on potato dextrose agar or Sorenson’s NP-10 semi-selective media. Genomic DNA from each culture (positive control) was extracted using E.Z.N.A.^®^ Fungal DNA Mini kit (Omega Bio-tek Inc., Norcross, GA, USA), and extracted DNA was dissolved in elution buffer solution (100 µL) and then stored at −20 °C until needed. PCR was conducted on unknown fungal cultures using the general fungal primers ITS1 and ITS4 [44]. A 25 µL solution consisting of 2.5 µL 2X Maxima Hot Start PCR Master Mix (Thermo Fisher Scientific, Waltham, MA, USA), 2.5 µL of 0.001 g/mL bovine serum albumin, 2 mM of magnesium chloride, 0.2 mM of dNTPs, 500 nM each of forward and reverse primer, and 0.1 µL/reaction of Maxima Hot Start taq (5U/µL) (Thermo Fisher Scientific, Waltham, MA, USA). The PCR program consisted of an initial denaturation cycle (94 °C, 3 min), followed by a denaturation, annealing, and extension cycle (94 °C for 30 s, 55 °C for 30 s, and 72 °C for 1.5 min) for 30 cycles, and finishing with a final extension period of 72 °C for 10 min. PCRs were conducted in an Eppendorf Mastercycler^®^ Nexus (Eppendorf, Mississauga, ON, Canada). PCR products were visualized on a 1.2% agarose gel to confirm the presence of the product. PCR products from *V. dahliae*, *V. albo-atrum*, and unknown cultures run with the ITS primers were purified using the QIAquick PCR Purification kit (Qiagen, Hilden, Germany). Purified products were quantified and assessed for purity on an Eppendorf BioPhotometer (Eppendorf, Mississauga, ON, Canada). All products were sent for sequencing along with their corresponding forward primer (Df, AaF, or ITS1; for the unknowns, ITS4 was also sent as the reverse) to the Centre for Applied Genomics (TCAG) (Toronto, ON, Canada). Results were compared to sequences available in GenBank using the Basic Local Alignment Search Tool (BLAST) of the Nucleotide Sequence Database (National Center for Biotechnology Information (NCBI), Bethesda, MD, USA) and species identity were confirmed.

Soil samples were also used to identify the species of nematodes within the genus of root-lesion nematodes. Standardized nematode morphometric techniques were used to identify *Pratylenchus* species at the Agriculture and Food Laboratory of the University of Guelph, Guelph, ON, Canada.

### 2.5. Verticillium Species Identification and Quantification Using RT-qPCR

*Verticillium dahliae* was detected in soil samples using RT-qPCR. DNeasy PowerSoil^®^ DNA Isolation Kits (Qiagen, Valencia, CA, USA) were used to extract DNA from each dried and sieved soil sample (250 mg). Three subsamples from each soil sample were extracted. Extracted DNA was dissolved in an elution buffer solution (100 µL) and stored at −20 °C. RT-qPCR was conducted using specific forward and reverse primers (not revealed for publication), targeting the elongation factor 1-alpha region of *V. dahliae*. A 20 µL solution consisting of 10 µL 2X Ssofast EvaGreen Supermix (Bio-Rad Laboratories, Mississauga, ON, Canada)), 2 µL of 0.001g/mL bovine serum albumin, species-specific primers of 400 nM forward and reverse [45], and template DNA (2 µL) was used to conduct an RT-qPCR. Reactions were run using the qTOWER3 real-time PCR system (Analytik Jena, Jena, Germany). The PCR program consisted of an initial denaturation cycle (98 °C, 2 min) followed by 45 cycles of denaturation (98 °C. 5 s) and species-specific annealing (10, 64 °C), extension (1 min, 72 °C). A melting curve analysis was conducted at the end of the program (60–95 °C) to confirm the absence of primer dimers and non-specific amplification products. The qPCRsoft (v. 3.2; Analytik Jena, Germany) was used to perform the assay sensitivity, measurements, and melting curve. Fungal genomic DNA was extracted from pure cultures using the E.Z.N.A.^®^ fungal DNA mini kit (Omega Bio-tek Inc., Norcross, GA, USA) and serially diluted (1:5, 5 ng/µL, 8 dilutions) to determine amplification efficiency and the standard curve. The comparative cycle threshold (CT) method was used to quantify pathogen DNA in each sample. Results were exported into Microsoft Excel where the standards were plotted (log of template concentration against CT values) and unknown amounts calculated through the generated curve equation. Three replicate reactions were conducted to assess the standard graph and sensitivity test. All ‘unknown’ samples were run in duplicate, and a positive soil control and no-template control (PCR grade water) were included on each 96-well plate. The amount of DNA corresponding to one *V. dahliae* cell was estimated at 36.5 fg/genome [46,47]. Results from RT-qPCR were used for pathogen identification and quantification (number of *V. dahliae* cells/g soil).

### 2.6. Evaluation of Chemical Fumigation (Chloropicrin)

Eight potato fields were randomly selected to assess the efficacy of chemical fumigation with Chloropicrin in managing PED pathogens. Chloropicrin was applied to the soil at the recommended rate of 55 L/ha in the fall as a conventional treatment. Each field was soil sampled before and after 3 weeks of fumigation. Soil samples were used to identify, determine, and quantify *V. dahliae* and *V. albo-atrum* to species level using plate count technique (CFU/g soil) at the Potato Development Centre, Wicklow, NB; RT-qPCR testing for *V. dahliae* (cell/g soil) was conducted at Agricultural Certification Services Inc., Fredericton, NB; and pathogenic nematode extraction and identification to genus level and counts (# of nematodes/kg of soil) using Baermann pan method were conducted at the Agriculture and Food Laboratory of the University of Guelph, Guelph, Ontario, as described earlier. The soil in the area from where samples were collected is silty clay loam to silty loam with >20% clay. Cation exchange capacity (CEC) in the region is 13 to 19 while the pH is maintained close to 6. Organic matter % ranged from 3.5 to 6%. Average air temperature during the months of May, June, July, August, and September were 10.9, 16.3, 18.7, 17.8, and 16.4 °C, respectively, with a total average of 16.0 °C. Total precipitation for the same months was 133.4, 68.8, 37.8, 38.0, and 57.2 mm, respectively, with a grand total of 335.2 mm.

### 2.7. Evaluation of Various Disease Control Products Against Nematodes and Verticillium spp.

A field-scale plot trial was conducted to test the efficacy of various products against *Verticillium* and root lesion nematode in a commercial field with a known history of PED. The field plot trial was laid out in a randomized block design (RBD) in the spring of 2017–2018 (see Section 2.6 for soil properties, precipitation, temperature, etc.). Soil samples were collected from all plots twice during the growing season: (1) 2 weeks after the potato crop was planted; and (2) at harvest. Soil samples were used for the identification, determination, and quantification of *Verticillium*, and pathogenic nematodes as described earlier. Nine treatments were applied pre-planting or at planting, and each treatment was replicated four times (4 strips). The treatments included the following:

- Control: No fungicide or nematicide applied.
- Aprovia: A fungicide applied in-furrow at the recommended rate for control of *Verticillium* wilt (0.625 L/ha; 100 L water/ha).
- Velum: A nematicide applied in-furrow at the recommended rate for control of pathogenic nematodes (4.5 mL/100m row; 100 L water/ha).
- Velum + Aprovia (Treatments #2 + #3) combined.
- Mustgrow: An “oriental mustard seed meal” that is surface applied and incorporated in the soil (1680.5 kg/ha).
- Senator PSPT: A “seed piece treatment” registered for the control of *Verticillium* wilt (500 g/100 kg of cut seed).
- Vapam: Soil fumigant for control of pathogenic nematodes applied in-furrow a minimum of 5 days prior to planting (487.5 L/ha).
- Ammonium-lignosulfonate: Applied as a soil amendment 14 days prior to planting, mixed with water applied directly to the soil (6 T/ha).
- Nimitz: Fast-acting contact nematicide for controlling root-knot and root lesion nematodes in fruiting vegetables. Applied in-furrow 7 days before planting at a depth of 15–20 cm (6 L/ha; 200 L water/ha).

Data were log transformed (log [x + 1]) before analysis. Due to similarities of data, the effects of treatments were examined by pooling data from two seasons. Statistical analyses were carried out, and field experiment data were subjected to analysis of variance (ANOVA) using the Statistix 9.0 software (Analytical Software, Tallahassee, FL, USA). If significant, a least significant difference was used as a mean separation test. Tuber yield, size distribution, and quality were assessed according to processing potato contracts. All differences are reported at *p* ≤ 0.05.

### 2.8. Field Visual Disease Assessment of Potato Early Dying (PED)

Twenty potato fields were visually assessed for *Verticillium* wilt disease severity based on PED symptoms. Disease incidence (number of plants exhibiting early dying symptoms out of 100 plants assessed per field) was assessed per potato field. Visual assessment was conducted by walking across the potato field following a ‘V’ pattern. For each 10 steps walked, a potato plant was assessed for *Verticillium* wilt symptoms, and disease incidence was calculated per field [48]. In addition, 10 potato plants suspected of *Verticillium* wilt infection were collected per field, and the stem parts were cultured and tested for the presence of *Verticillium.* Stems were placed in 1.5–1.7% calcium hypochlorite solution for 2 min, then in sterile distilled water for 2 min, and then on sterilized filter paper. Tissues were cut into 0.2–0.5 cm pieces using sterile scalpel and tweezers and then transferred onto NP-10 media. Plates were incubated for 1–2 weeks at room temperature before they were examined for the presence of *Verticillium* MS under a dissecting microscope. *Verticillium* cultures were purified, identified, and sequenced using conventional PCR.

## 3. Results

### 3.1. Plant and Soil Survey for Verticillium Species and Nematodes in NB Potato Fields

**Figure 2:**
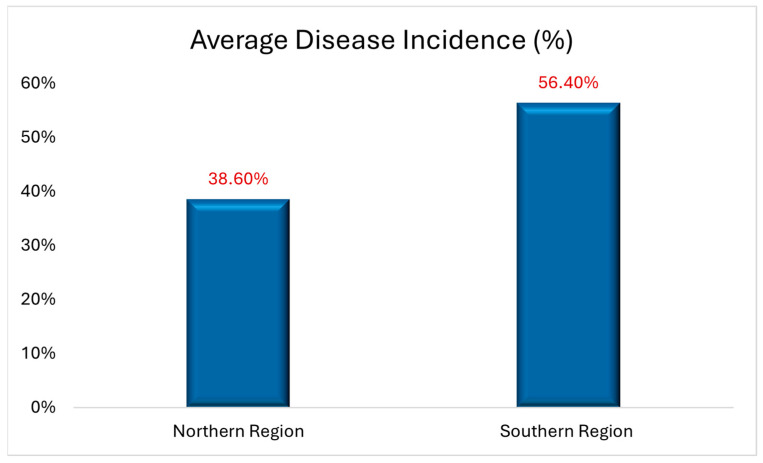
Average disease incidence of potato early dying (PED) based on visual wilt symptoms in 10 fields in the northern region and 10 fields in the southern region of the potato belt area of New Brunswick, Canada.

**Figure 3:**
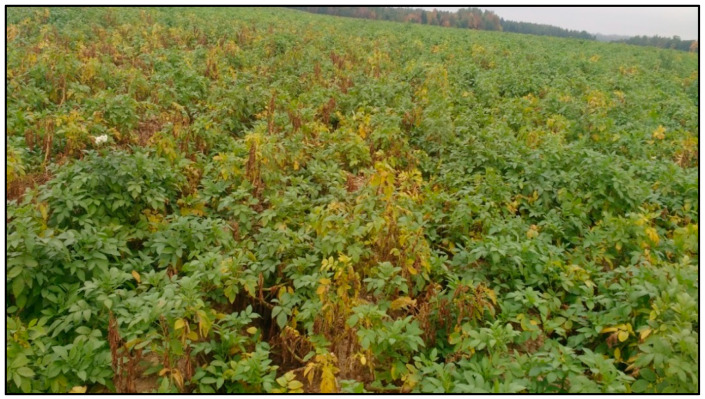
Visual assessment of potato early dying (PED) symptoms of potato fields in the northern region of the potato belt of New Brunswick, Canada.

**Figure 4:**
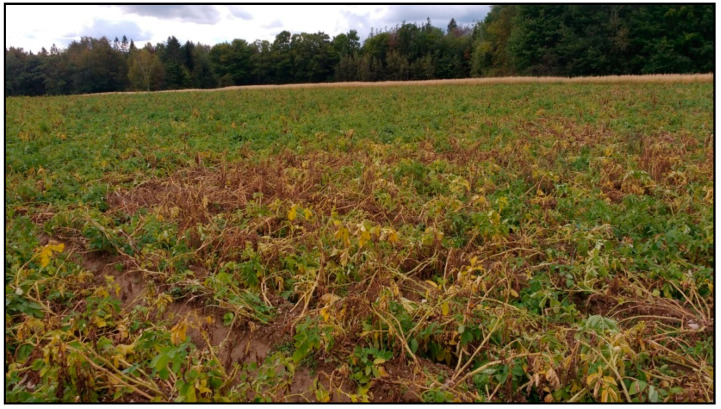
Visual assessment of potato early dying (PED) symptoms of potato fields in the southern region of the potato belt of New Brunswick, Canada.

**Figure 5:**
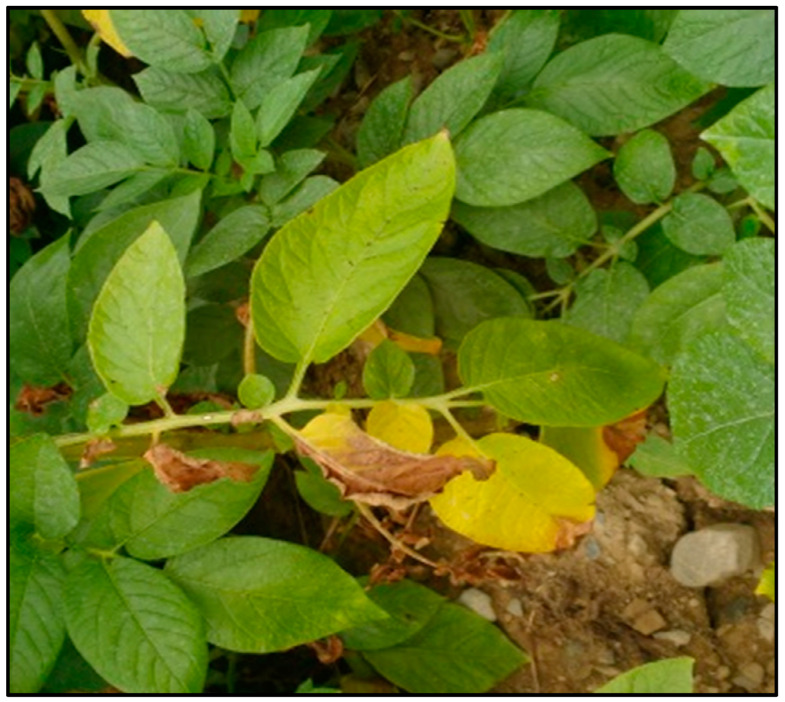
Potato leaves exhibiting *Verticillium* wilt symptoms of yellowing and necrosis.

Disease incidence assessments of PED based on visual wilt symptoms (100 plants/field) were made in 20 NB potato fields (10 in the northern zone and 10 in the southern zone of the potato growing regions of NB) where severe early dying symptoms were observed in September 2017. Results revealed higher disease incidence (56.4%) in fields in the south compared to those in the north (38.6%) (Figure 2, Figure 3 and Figure 4). All potato plants collected from PED-suspected fields tested positive for *V. dahliae* (Figure 5). *V. dahliae* was identified in all collected potato plants (100% occurrence in each of the 20 fields) with 98–99% sequence similarity to the deposited sequences in NCBI-GeneBank. These results confirmed that *V. dahliae* is the main contributor to the early dying symptoms.

**Figure 6:**
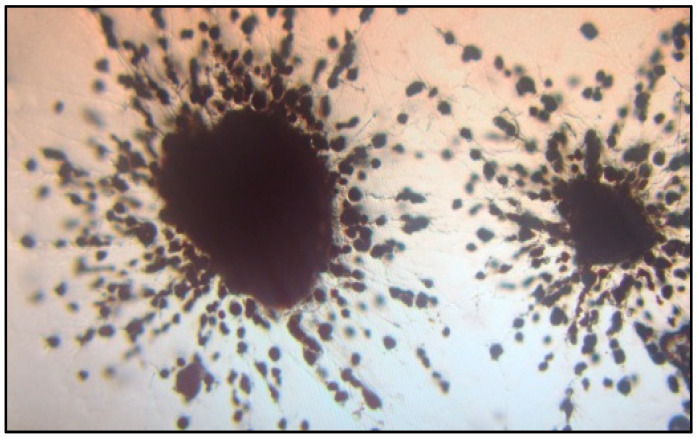
Microsclerotia of *Verticillium* spp. grown on Sorenson’s NP-10 semi-selective medium inspected under dissecting microscope (100×).

**Figure 7:**
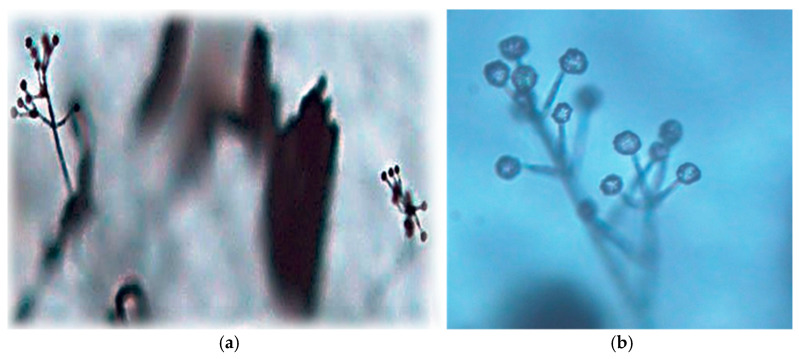
*Verticillium* spp. conidiophore and conidia under a light microscope: (**a**) low-power magnification (40×); (**b**) high-power magnification (400×).

A survey of 71 commercial potato fields was conducted in NB in the fall of 2017 to determine the presence of *V. dahliae* and nematodes. The survey of the incidence and pathogen/nematode abundance was carried out using plate counts, RT-qPCR, and nematode identification and counts. High levels of *Verticillium* wilt pathogens and root-lesion nematodes were detected in many fields. Approximately 77.5% of the surveyed fields tested positive for *Verticillium* spp. microsclerotia (Figure 6 and Figure 7), with a maximum number of 66 CFU/g of soil (Table 1). Approximately 42% of the fields had 2–13 CFU/g soil, and 5.6% of the fields had >30 CFU/g when quantified using the plate count method. The economic damage threshold level for *V. dahliae* alone and in the presence of nematodes is 5–30 and 2–13 CFU/g of soil, respectively, as previously reported [1]. RT-qPCR analysis of DNA extracted from 71 field soil samples revealed that the incidence of *V. dahliae* in NB was high with an uneven distribution. Analysis of the 71 composite soil samples also confirmed positive results for *V. dahliae* and wide distribution in NB fields, occurring with a high frequency (100% incidence) in 68 fields cultivated with potatoes. There were only three soil samples from which no *V. dahliae* DNA was detected. The abundance of *V. dahliae* varied largely in 68 field soil samples c and ranged from 261 to 27,471 cell/g soil. The heaviest infestation was recorded in the parish of Grand Falls, field #NV2017-022, with an average of 27,471.4 *V. dahliae* cells/g of soil (Table 1).

Five nematode genera, namely, root lesion nematode (*Pratylenchus* spp.), pin nematode (*Paratylenchus* spp.), spiral nematode (*Helicotylenchus* spp.), root knot nematode (*Meloidogyne* spp.), or stunt nematode (*Tylenchorhynchus* spp.), were identified in soils from NB fields in 2017 (Table 1). The economic damage threshold level for root lesion nematodes is 1000/kg of soil for susceptible cultivars such as “Superior” and 2000/kg of soil for all other cultivars ( (https://cropipm.omafra.gov.on.ca/en-ca/crops/potatoes/diseases/17daf253-bd84-486b-a2b3-7a40a93044c0?iid=0c956cd4-cfc5-4561-8518-7a6efc21df32, accessed on 30 March 2025).

Results indicated that 90.2% of the fields were above the 1000/kg soil economic damage threshold for root lesion nematode and 63.4% above the 2000/kg soil economic damage threshold with a maximum number of root lesion nematodes of 14,240/kg soil. Data in Table 2 illustrate the total number of soil samples collected from both northern (26 samples) and the southern (45 samples) regions of the potato belt area of NB containing all five nematode species. All 71 soil samples collected in 2017 from both regions contained root lesion nematode (*Pratylenchus* spp.) and pin (*Paratylenchus* spp.). A small number of soil samples had other parasitic nematodes, such as root knot (*Meloidogyne* spp.), stunt (*Tylenchorhynchus* spp.), and spiral (*Helicotylenchus* spp.) nematodes (Table 2).

### 3.2. Effect of Fumigation with Chloropicrin on Root Lesion Nematode and Verticillium dahliae Population Density in Soil of New Brunswick Fields in the Fall of 2017

Eight potato fields were randomly selected in 2017 to assess the efficacy of chemical fumigation (Chloropicrin) in managing potato early dying disease. In comparison with non-fumigated fields, fumigation with Chloropicrin had a greater effect on reducing the population density of the root lesion, pin, root-knot, and spiral nematodes. Five fields showed more than 90% reduction in root lesion nematode. A maximum reduction in root lesion nematode was recorded in field #2 (99%), and the lowest reduction was in field #3 (34%) compared to the same fields when tested before fumigation (Table 3). In addition, fumigation with Chloropicrin had a greater effect on reducing the population density of *Verticillium* spp. (CFU/g soil) in soil samples collected from fumigated fields compared to before fumigation. Four fields showed a 100% reduction in *Verticillium* spp. (CFU/g soil), and four fields showed moderate reduction ranging from 50% to 90% (CFU/g soil) (Table 3). Fumigation with Chloropicrin showed greater effectiveness in controlling *V. dahliae* in the fumigated field soil samples compared to non-fumigated samples. Seven fumigated field soil samples showed the highest reduction in the population density of *V. dahliae* ranging from 80 to 91% (cells/g of soil). Only field #3 showed less effect on the population density of *V. dahliae* with 37.9% (cell/g of soil), as illustrated in Table 3.

### 3.3. Evaluation of Various Disease Control Products Against Nematodes and Verticillium (Field Plot Trial)

Various disease control products such as Aprovia (fungicide registered for the management of *Verticillium*), Velum (nematicide), Mustgrow (mustard seed meal), Senator PSPT (seed piece treatment fungicide), Vapam (nematicide, fumigant), Nimitz (contact nematicide), and ammonium-lignosulfonate (soil amendment) were used to manage potato early dying and to test their efficacy against root lesion nematode and *Verticillium* population density under field conditions in 2017–2018. Results (Table 4) indicated a variation in the effectiveness of the different disease control products against root lesion nematode and *Verticillium* population density compared to the untreated control. Treatments with the nematicide Velum, Velum + Aprovia, Vapam, and ammonium-lignosulfonate had greater effects on reducing the population density of the root lesion nematode (66.67%, 40.5%, 25.0%, and 32.7%, respectively) compared to the untreated control and other products (Table 4). The efficacy of various disease control products against *Verticillium* spp. (CFU/g soil) under field conditions is illustrated in Table 5. Most of the treatments caused a reduction in *Verticillium* spp. counts (CFU/g soil), which ranged from 16.1% to 73.2% compared to the untreated control. The fungicide Aprovia applied in-furrow at the recommended rate for the management of *Verticillium* wilt significantly reduced *Verticillium* spp. CFU/g soil by 73.3% compared to other products and the untreated control. Treatments with Senator PSPT and Nimitz had no effect on *Verticillium* population density (Table 5). All treatments except that with Nimitz showed efficacy in reducing *V. dahliae* content (cell#/g soil). The combination of Velum + Aprovia and the application of ammonium-lignosulfonate significantly reduced *V. dahliae* by 190.95% and 274.24%, respectively, compared to the untreated control and other products (Table 6). All tested products increased potato marketable yield by 27.38–97.74% compared to the untreated control (Table 7).

## 4. Discussion

Potato early dying (PED) disease complex causes loss in vigor during mid to late summer, followed by premature vine senescence, resulting in mild to severe tuber yield losses and consequently significant crop yield reduction by more than 50% [1,23,47,49,50]. Fungal wilt pathogens such as *Verticillium dahliae* and *V. albo-atrum* are considered the main causal agents of PED disease, and they exhibit different levels of aggressiveness [49,51]. The fungus can overwinter in infected tubers and plant debris. It can spread from one part of the field to another by runoff water, cultivation, and machinery such as harvesters. Soil parasitic nematodes [52], such as root-lesion nematode (*Pratylenchus penetrans*), is also an important factor contributing to the PED disease incidence and yield loss. The co-infection of potato by both *V. dahliae* and the root lesion nematode can greatly increase the severity of the disease.

Our soil survey was designed to study the distribution and levels of nematodes and *Verticillium* pathogens in commercial fields cultivated with potatoes in NB, Canada, in 2017. Results illustrated the presence of five soil parasitic nematodes, particularly root lesion nematode (*Pratylenchus* spp.), in all 71 fields. More than 77% of the surveyed fields had *Verticillium* spp. when quantified using the plate count method, and 96% of the surveyed fields had *V. dahliae* when quantified using an RT-qPCR technique with an uneven distribution in the various locations of NB. Our results were similar to those reported by other researchers [27,45,53,54] who reported the presence of *V. dahliae* in all potato-growing regions of Canada. Root-lesion nematode (*P. penetrans*) was detected in central Canada, the Maritimes, and British Columbia. Our results demonstrated that more than 90% of the fields under study in NB were above the economic damage threshold of 1000/kg soil for root lesion nematode (https://cropipm.omafra.gov.on.ca/en-ca/crops/potatoes/diseases/17daf253-bd84-486b-a2b3-7a40a93044c0?iid=0c956cd4-cfc5-4561-8518-7a6efc21df32 (accessed on 30 March 2025) with a maximum number of 14,240/kg of soil. This is evidence of a much greater issue associated with potato early dying than previously thought, so there is a need to better address this issue in future studies. These results are comparable with the outcomes of several investigations conducted in Canada and other countries [7,9,10,11,23,24,25,26,45,51,55,56]. *Verticillium* wilt, a soil-borne disease of potato, is difficult to control due to the longevity of microsclerotia, broad host range, and the fact that none of the potato cultivars are completely resistant or tolerant to *V. dahliae*; therefore, control strategies of *Verticillium* wilt in Canada are generally limited to a combination of chemical and cultural practices such as crop rotations and the use of resistant cultivars. As a result, an adequate PED disease management strategy should consider the integration of different potential protection options, including soil fumigation with metam sodium (Vapam) or Chloropicrin, alone or in combination with other chemicals [7,35,57,58]. PED management tactics should be aimed at preventing the elevation of *Verticillium* wilt inoculum in soil. Chemical fumigants are very effective in killing the root lesion nematode. However, a reduction in *Verticillium* soil levels is more difficult to achieve. Fumigation with Chloropicrin had a greater effect on reducing the population density of the root lesion nematode by 99%; *Verticillium* spp. by 100% (CFU/g soil); and *V. dahliae* by up to 91% (cells of *V. dahliae*/g soil) in soil samples collected after fumigation compared to those collected from the same fields before fumigation. The reduced effectiveness of fumigation with Chloropicrin in field # 3 may be due to several factors, such as a lower application rate compared to maximum rates used in potato growing regions in Canada, low concentration of active ingredient used compared to other commercial formulations, dry soil conditions at the time of application, water content in the soil, survival of the pathogen deeper in the soil, and weather factors, etc. However, our results showed a similar trend to other studies [7,57,58,59].

Chemical fumigation is an effective method to manage PED. Effective chemical fumigation represents a significant input cost for growers. It may also adversely affect soil health. Consequently, it is necessary to consider alternatives to chemical fumigation for managing PED. Therefore, host resistance is the best means of controlling PED [60,61]. We studied the efficacy of alternative products to fumigation against nematodes and *Verticillium* using a field with known high levels of both *Verticillium* and root lesion nematode in 2016. Treatments with Velum, Vapam, Velum + Aprovia, and ammonium-lignosulfonate had greater effects on reducing the population density of the root lesion nematode compared to other products. Most of the treatments showed a good reduction in *Verticillium* spp. counts (CFU/g soil) compared to the untreated control. The fungicide Aprovia was highly effective in reducing *V. dahliae* CFU/g of soil in treated soil by 73.3% compared to other products and the untreated control. The combination of Velum + Aprovia and the application of ammonium-lignosulfonate significantly reduced *V. dahliae* by 190.95% and 274.24%, respectively, compared to other products. All disease management products increased potato marketable yield by 27.38–97.74%. Historically, potato yields in New Brunswick tend to be lower than Western Canada and USA, as we do not rely on irrigation due to topography limitations. Our results agree with other studies. Kirkegaard et al. [62] used cruciferous crop residues as biofumigants to manage PED. Mustard reduced soil-borne diseases of potatoes more than other rotation crops [28,63]. In Eastern Canada, high glucosinolate mustard varieties were used as biofumigants to manage PED [64]. Biofumigation has been shown to reduce nematode populations in potato fields [65,66]. Brassica green manure [67] and crop rotation are well known to reduce disease pressure and increase potato yields. Marigolds [47] and pearl millet [63,68] have shown good potential to reduce the impact of PED on potatoes. Our study revealed that the management practices of PED using the fungicide Aprovia and nematicide Velum and a combination of both Velum + Aprovia had the greatest effect in reducing the population density of the root lesion nematode and *V. dahliae* in soil.

## 5. Conclusions

The results of this study suggest that root lesion nematode and *V. dahliae* have a ubiquitous distribution in the fields cultivated with potatoes in NB and are consistent with similar studies carried out in Nova Scotia [54,56] and Prince Edward Island [45]. The co-infection of potato by both *V. dahliae* and the root lesion nematode can greatly increase the severity of PED. Fumigation with Chloropicrin significantly managed PED by reducing the levels of root lesion nematode and *Verticillium* in all fumigated fields. The management practices of PED using the fungicide Aprovia and nematicide Velum and a combination of both Velum + Aprovia had the greatest effect in reducing the population density of the root lesion nematode and *Verticillium dahliae* in soils of commercial potato fields in New Brunswick.

For future research, the authors invite scholars to perform similar province-specific studies focusing on the impact of climate change on the population density of *Pratylenchus penetrans* and *Verticillium* species and their impact on PED symptoms and potato marketable yield. Testing alternative management approaches and returns on investment studies are also advised. Such academic contributions can help achieve better management of PED.

## Figures and Tables

**Table 1:** Results of soil testing for nematodes (#/kg soil) and *Verticillium* (cell/g of soil and CFU/g of soil) for soils collected from 71 New Brunswick (Canada) potato fields in 2017.

Field #	Sample ID	Parish	Nematode #/kg of Soil					*Verticillium* Content	
			Root-Lesion	Pin	Root-Knot	Stunt	Spiral	# V. d * Cells/g	V. w ** CFU/g
1	VN2017-001	Drummond	5320	5920	0	0	0	3234.6	8
2	VN2017-002	Drummond	8720	0	20	0	0	13,991.1	20
3	VN2017-003	Drummond	5720	0	0	0	0	1866.7	6
4	VN2017-004	Drummond	5000	0	0	0	0	11,489.8	30
5	VN2017-005	Drummond	3640	80	0	40	0	3382.2	2
6	VN2017-006	Drummond	6360	0	0	0	0	261.4	16
7	VN2017-007	Drummond	4800	40	0	0	0	1267.5	36
8	VN2017-008	St-André	1820	20	0	0	0	19,688.5	34
9	VN2017-009	St-André	5080	240	0	0	0	2480.9	14
10	VN2017-010	St-André	1160	0	0	0	0	4254.9	14
11	VN2017-011	St-André	4620	0	0	0	0	1876.1	12
12	VN2017-012	Grand Falls	3820	20	0	0	0	2287.7	10
13	VN2017-013	Grand Falls	9400	0	0	0	0	4126.0	14
14	VN2017-014	New Denmark	2120	0	0	0	0	814.7	10
15	VN2017-016	New Denmark	1080	140	0	0	0	1364.4	12
16	VN2017-017	New Denmark	5320	260	0	0	20	0.0	2
17	VN2017-018	Four Falls	4400	160	0	0	100	3489.6	10
18	VN2017-019	Four Falls	1480	4320	0	0	180	4359.2	2
19	VN2017-020	St-André	580	0	0	0	300	423.1	24
20	VN2017-021	St-André	5460	120	560	0	660	1294.3	0
21	VN2017-022	Grand Falls	3580	0	0	0	0	27,471.4	14
22	VN2017-023	Grand Falls	5960	40	0	0	0	3704.2	8
23	VN2017-024	Grand Falls	7320	0	0	0	0	6025.8	16
24	VN2017-025	St-André	680	0	120	0	400	18,147.0	16
25	VN2017-026	St-André	13,640	0	0	0	0	4304.3	6
26	VN2017-027	Greenfield	1100	100	0	0	0	16,287.5	0
27	VN2017-028	Greenfield	10,680	0	0	0	0	2557.6	2
28	VN2017-029	Jacksonville	2680	360	0	0	0	16,487.5	0
29	VN2017-030	Jacksonville	2160	280	0	0	40	943.7	2
30	VN2017-032	St-Thomas	4260	0	0	0	0	2583.9	0
31	VN2017-033	St-Thomas	6600	740	20	0	180	2529.7	16
32	VN2017-034	Simonds	2180	20	0	0		3291.6	6
33	VN2017-036	Knoxford	1080	60	0	0	20	4971.1	0
34	VN2017-038	Jacksonville	1540	0	660	0	0	13,623.5	8
35	VN2017-039	Jacksonville	8520	0	0	0	0	4589.5	4
36	VN2017-041	Killoween	3680	320	1320	0	0	3193.4	0
37	VN2017-042	Bloomfield	10,760	0	0	0	0	443.4	0
38	VN2017-043	Bloomfield	14,240	0	0	0	0	1839.5	14
39	VN2017-044	Hartland	1900	40	0	0	0	21,503.0	66
40	VN2017-045	Hartland	8400	2600	0	0	0	1350.7	2
41	VN2017-046	Connell	1580	0	0	0	0	10,287.6	24
42	VN2017-047	Connell	2380	0	0	0	0	1390.5	10
43	VN2017-048	Richmond Corner	2740	20	0	0	100	3027.5	6
44	VN2017-049	Richmond Corner	1400	0	0	0	0	2797.5	12
45	VN2017-050	Grand Falls	9840	0	0	0	0	3793.2	0
46	VN2017-051	Knoxford	1400	20	1080	0	680	0.0	0
47	VN2017-052	Wicklow	1080	60	0	0	20	4971.1	0
48	VN2017-053	Wakefield	560	0	0	0	0	0.0	2
49	VN2017-054	Wakefield	1480	0	0	0	0	4838.0	0
50	VN2017-055	Wakefield	1120	0	0	0	40	5282.7	4
51	VN2017-056	Wakefield	3360	60	0	0	0	1703.2	10
52	VN2017-057	Wakefield	2300	120	0	0	0	3259.8	0
53	VN2017-058	Wakefield	4520	0	0	0	0	5427.2	18
54	VN2017-059	Wicklow	820	0	0	0	0	7894.1	0
55	VN2017-060	Wicklow	820	0	0	0	0	7894.1	0
56	VN2017-061	Wakefield	1580	0	0	0	20	7600.1	6
57	VN2017-062	Wicklow	1180	0	0	0	80	3734.0	0
58	VN2017-063	Wicklow	1780	0	0	0	20	1690.8	6
59	VN2017-064	Simonds	900	0	0	0	0	13,737.5	18
60	VN2017-065	Aberdeen	1480	0	0	0	20	8368.3	4
61	VN2017-066	Wakefield	3840	60	0	0	0	6075.3	18
62	VN2017-067	Aberdeen	1100	20	0	0	0	24,055.6	34
63	VN2017-068	Simonds	3840	0	0	0	40	6159.4	20
64	VN2017-069	Simonds	9080	0	0	0	20	2178.1	26
65	VN2017-070	Wilmot	6160	0	0	0	0	1848.8	2
66	VN2017-071	Wilmot	620	0	0	0	0	2438.0	0
67	VN2017-072	Wilmot	5580	0	0	0	0	7705.0	24
68	VN2017-073	Wakefield	2500	0	0	0	60	1564.4	14
69	VN2017-074	Wilmot	6720	0	0	0	0	303.3	4
70	VN2017-075	Wilmot	3960	0	0	100	0	1788.3	10
71	VN2017-076	Wakefield	8000	0	0	0	0	1042.2	20

Field # represents the field from which samples were collected. * V. d: Verticillium dahliae and ** V. w: Verticillium wilt.

**Table 2:** Number of soil samples containing root lesion (*Pratylenchus* spp.), pin (*Paratylenchus* spp.), root knot (*Meloidogyne* spp.), stunt (*Tylenchorhynchus* spp.), or spiral (*Helicotylenchus* spp.) nematodes out of 71 soil samples collected from potato fields in various parishes of New Brunswick, Canada, in the fall of 2017.

Parish	No. of Samples	No. of Soil Samples Containing Nematodes				
		Root Lesion	Pin	Root-Knot	Stunt	Spiral
Drummond	12	12	3	1	1	0
Grand Falls	8	8	4	0	0	2
St-André	8	8	3	2	0	3
Brighton	2	2	2	0	0	0
Kent	1	1	1	1	0	0
Richmond	2	2	1	0	0	1
Simonds	9	9	3	1	0	4
Wakefield	11	11	3	0	0	3
Wicklow	9	9	4	1	0	5
Wilmot	9	9	1	1	1	0

**Table 3:** Effect of fumigation with Chloropicrin on root lesion nematode (*Pratylenchus* spp.) and *Verticillium* population density in soils from potato fields in New Brunswick, Canada, in the fall of 2017.

Sample ID	Nematode (#/kg Soil)									*Verticillium* Content/g Soil					
	Root Lesion			Pin		Root-Knot		Spiral		# V. d *^a^ Cells/g			V. w ** (CFU/g)		
	*** BF	**** AF	% Control	BF	AF	BF	AF	BF	AF	BF	AF	% Control	BF	AF	% Control
Field 1	2500	140	94.4	0	0	0	0	0	0	10,837 ± 4504	962.8 ± 324	91.1	8.0	0	100.0
Field 2	4120	40	99.0	0	0	0	0	0	0	10,398.2 ± 2302	1968.4 ± 1053	81.1	10.0	1	90.0
Field 3	11,080	7300	34.1	280	20	0	0	600	0	807.6 ± 387	501.5 ± 130	37.9	4.0	2	50.0
Field 4	8520	1160	86.4	80	0	0	0	200	0	797.4 ± 115	156.3 ± 93	80.4	6.0	1	83.3
Field 5	3120	140	95.5	0	0	0	0	0	0	830.3 ± 382	38.1 ± 18	95.4	8.0	2	75.0
Field 6	2040	200	90.2	80	0	0	0	20	0	1736.4 ± 209	220.1 ± 125	87.3	6.0	0	100.0
Field 7	780	280	64.1	380	80	0	0	20	0	4291.3 ± 740	536.8 ± 251	87.5	6.0	0	100.0
Field 8	3660	140	96.2	80	0	20	0	0	0	14,412.8 ± 7682	1567.3 ± 550	89.1	14.0	0	100.0

* V. d: *Verticillium dahliae*; ** V. w: *Verticillium* wilt; *** BF: before fumigation; and **** AF: after fumigation. ^a^ Values ± standard errors are the average of three replicates.

**Table 4:** Evaluation of various potato disease management products against root lesion nematode (*Pratylenchus* spp.), under field conditions in New Brunswick, Canada.

Treatment	Root Lesion Nematode (#/kg Soil) *		% Increase (+)/Decrease (−) of Root Lesion Nematodes **
	Spring Samples	Fall Samples	
Control	190	365	+92.11 a
Aprovia	265	290	+9.43 d
Velum	450	150	−66.67 h
Velum + Aprovia	605	360	−40.50 g
Mustgrow	360	480	+33.33 c
Senator PSPT	585	675	+15.38 d
Vapam	440	330	−25.00 f
Ammonium-lignosulfonate	765	515	−32.68 fg
Nimitz	505	510	+0.99 e

* Values are the average of four replicates. Data are average values of 2 seasons combined. ** Means followed by the same letter in the same column are not significantly different (*p* ≤ 0.05).

**Table 5:** Evaluation of various potato disease management products against *Verticillium dahliae* (CFU/ g soil) under field conditions in New Brunswick, Canada.

Treatment	*Verticillium dahliae* CFU/g Soil *		% Increase (+)/Decrease (−) of CFU/g Soil **
	Spring Samples	Fall Samples	
Control	4.5	20.0	+344.4 a
Aprovia	7.5	2.0	−73.3 c
Velum	46.0	27.5	−40.0 c
Velum + Aprovia	15.5	13.0	−16.1 c
Mustgrow	25.5	14.5	−43.1 c
Senator PSPT	19.5	12.0	−38.5 c
Vapam	11.5	9.5	−17.4 c
Ammonium-lignosulfonate	7.50	3.5	−53.3 c
Nimitz	4.50	13.3	+195.6 b

* Values are the average of four replicates. Data are average values of 2 seasons combined. ** Means followed by the same letter in the same column are not significantly different (*p* ≤ 0.05).

**Table 6:** Evaluation of various potato disease management products against *Verticillium dahliae* (cell#/g soil) under field conditions in New Brunswick, Canada.

Treatment	*Verticillium dahliae* Content Cell#/g Soil *		% Increase (+)/Decrease (−) Cell#/g Soil **
	Spring Samples	Fall Samples	
Control	820.3	3840.1	+78.64 a
Aprovia	6825.6	3914.8	−74.35 b
Velum	2301.4	1517.5	−51.66 b
Velum + Aprovia	9787.4	3368.6	−190.55 d
Mustgrow	2281.1	2088.6	−9.22 b
Senator PSPT	3102.3	2831.0	−9.58 b
Vapam	2272.5	997.0	−127.93 c
Ammonium-lignosulfonate	4917.3	1313.9	−274.24 e
Nimitz	1151.5	3477.7	+66.89 a

* Values are the average of four replicates. Data are average values of 2 seasons combined. ** Means followed by the same letter in the same column are not significantly different (*p* ≤ 0.05).

**Table 7:** Effect of various potato disease management products on potato marketable yield (T/ha), New Brunswick, Canada.

Treatment	Marketable Yield (T/ha) *	% Increase Relative to Untreated Control
Control	13.62 c	0.00
Aprovia	22.89 ab	67.99
Velum	25.09 ab	84.12
Velum + Aprovia	19.95 abc	46.41
Mustgrow	25.06 ab	83.91
Senator PSPT	22.74 ab	66.89
Vapam	17.36 bc	27.38
Ammonium-lignosulfonate	26.66 a	95.67
Nimitz	26.94 a	97.74

* Values are the average of four replicates. Data are average values of 2 seasons combined. Means followed by the same letter in the same column are not significantly different (*p* ≤ 0.05).

## Data Availability

The original contributions presented in this study are included in the article. Further inquiries can be directed to the corresponding author.
